# Ecotoxicological Assessment of Landfill Leachate Treated by Coagulation-Flocculation-Sedimentation and Powdered Activated Carbon Adsorption

**DOI:** 10.1007/s00128-026-04250-x

**Published:** 2026-06-16

**Authors:** Jandiara Damaris Campos Pozzetti, Renan Borelli Galvão, Vilson Gomes da Assunção Júnior, Emília Kiyomi Kuroda

**Affiliations:** 1 Coordenação de Curso de Engenharia Civil, Universidade Tecnológica Federal Do Paraná, Rua Marcílio Dias 635, Jardim Paraiso, Apucarana, PR 86812460 Brazil; 2 Departamento de Construção Civil, Centro de Tecnologia E Urbanismo, Universidade Estadual de Londrina, Rodovia Celso Garcia Cid, PR445, Km380, Londrina, PR 86057970 Brazil

**Keywords:** Ecotoxicity, Landfill leachate, Physicochemical treatment, Powdered activated carbon

## Abstract

The isolated evaluation of physicochemical parameters of effluents in landfill leachate treatment is insufficient to determine reductions in acute or chronic toxicity to aquatic biota. Therefore, ecotoxicity tests are essential to assess environmental contamination. This study evaluated the ecotoxicological impacts, through acute and chronic tests on different organisms, of raw landfill leachate, leachate treated with ammonia stripping and activated sludge, and leachate post-treated by coagulation–flocculation–sedimentation (CFS) and adsorption using powdered activated carbon (PAC). Tests with *P. subcapitata*, *C. dubia*, *D. magna*, and *A. salina* indicated that biologically treated leachate exhibited lower toxicity than raw leachate. However, during post-treatment with CFS combined with PAC adsorption, despite the significant removal of recalcitrant compounds, toxicity increased for *P. subcapitata*, *C. dubia*, and *D. magna*, likely due to residual metals and chlorides from the coagulant and acidifier. Among the tested organisms, *A. salina* exhibited responses that differed from those of the other species, with a gradual decrease in toxicity observed after each treatment stage. This behavior may be partially associated with its marine origin and potential tolerance to chlorides.

## Introduction

Sanitary landfills are the most common method for the final disposal of solid waste in Brazil (Alfaia et al. 2017; Costa et al. 2019). However, this disposal method requires measures to protect the local environment and its surroundings due to its potential impacts, particularly from biogas generation and leachate production.

In landfills, the physical, chemical, and biological decomposition of organic matter, combined with the percolation of rainwater, generates leachate, a dark liquid with an unpleasant odor and high polluting potential. This effluent has a complex composition, including high concentrations of ammoniacal nitrogen, organic and inorganic matter, recalcitrant organic compounds (e.g., humic substances), metals, and other toxic substances (Chen et al. 2023; Renou et al. 2008; PSG da Silva et al. 2022; Vaccari et al. 2019).

Proper treatment of leachate is essential to comply with legal limits for discharge into water bodies and to minimize environmental impacts. Such treatments typically combine biological and physicochemical processes (Babaei et al. 2021; Bashir et al. 2017; Chaouki et al. 2017; Erabee et al. 2018; Mahdavi et al. 2018; Modin et al. 2011; Verma et al. 2023).

The treatment sequence investigated in this study reflects a strategy applicable to the management of stabilized landfill leachate. Ammonia stripping is typically employed to reduce high ammoniacal nitrogen concentrations that may inhibit biological processes. Subsequent activated sludge treatment promotes organic matter biodegradation, while coagulation–flocculation–sedimentation (CFS) and powdered activated carbon (PAC) adsorption are applied as polishing steps to remove recalcitrant organic compounds and residual toxic constituents.

However, leachate treatment techniques do not always ensure toxicity removal. Traditional physicochemical characterization cannot distinguish between substances that are biologically active and those inert to the environment. Therefore, it is critical to control effluent quality to prevent toxic effects, whether acute or chronic, on aquatic biota. In this regard, several studies have focused on the ecotoxicological assessment of landfill leachate (Bastos et al. 2021; Escalante-Mañe et al. 2022; Gallão et al. 2019; Grilla et al. 2024; Pablos et al. 2011; Sackey et al. 2020; Storck et al. 2023; Tigini et al. 2014).

Although numerous studies have evaluated landfill leachate treatment processes, most investigations focus on isolated treatment steps or assess toxicity only before and after a single process (Da Costa et al. 2018; De Sousa et al. 2023; Fernandes et al. 2019; Gamoń et al. 2019; Hussain et al. 2023). Limited information is available regarding how toxicity evolves throughout sequential treatment stages and whether combined physicochemical post-treatment processes effectively mitigate both acute and chronic effects.

Ecotoxicity tests are valuable tools for evaluating environmental contamination from various pollution sources. They assess the toxic potential of substances or mixtures on living organisms and measure the extent of their harmful effects. These tests also offer the advantage of analyzing a wide range of biologically available substances present in environmental samples (Magalhães and Ferrão Filho 2008).

Updates to Brazilian environmental legislation have incorporated ecotoxicity testing (e.g., CONAMA 357/2005; CONAMA 430/2011; CEMA No. 0070/2009; CEMA No. 081/2010). Thus, to preserve aquatic environments, it is crucial to assess both the toxicity of leachate effluents from different treatment stages and their physicochemical parameters to evaluate system performance comprehensively.

In this context, the present study aimed to perform an integrated ecotoxicological assessment, using both acute and chronic bioassays with organisms representing different trophic levels, of raw landfill leachate and leachate subjected to sequential treatment stages: preliminary ammonia stripping followed by biological treatment with activated sludge; post-treatment by CFS; and post-treatment by CFS combined with PAC adsorption. Particular attention was given to determining whether the addition of physicochemical polishing steps enhances toxicity removal compared to biological treatment alone, thereby contributing to improved environmental safety of the treated effluent.

## Methods and Materials

### Biological and Physicochemical Treatments

To evaluate the potential reduction in toxicity during the treatment of landfill leachate, the following sequential treatment steps were applied to the raw leachate: (i) preliminary treatment through ammonia stripping followed by biological treatment using activated sludge; (ii) post-treatment via coagulation-flocculation-sedimentation; and (iii) post-treatment via coagulation-flocculation-sedimentation combined with adsorption using pulverized activated carbon.

The raw leachate was collected from the municipal solid waste landfill located in Londrina, Paraná, Brazil. This landfill operated from 1974 to 2010, and the collected leachate exhibits characteristics consistent with stabilized leachate.

Initially, the raw leachate underwent pretreatment through ammonia stripping, followed by biological treatment using activated sludge. The ammonia stripping pretreatment was conducted in a sequential batch system comprising two reactors, each with a volumetric capacity of 1 m^3^. These reactors were equipped with rotating polyethylene and stainless-steel paddles, operating at an approximate rotation speed of 5.5 rpm over a 20-day period. The biological treatment using activated sludge was performed in a 2 m^3^ reactor. This treatment included an aerobic phase, during which aeration was achieved using an air compressor and diffusers constructed from perforated PVC tubing. Following the aerobic phase, an anoxic phase was implemented, during which ethanol was added as a carbon source, dosed based on residual nitrite and nitrate concentrations, to enable short denitrification and promote additional removal of nitrogen compounds.

Samples of raw leachate and biologically treated leachate were collected directly from the reactor tanks immediately before the start of ammonia stripping and after completion of the activated sludge process, respectively. Sampling was carried out after homogenization of the leachate, with a total volume of 5 L collected.

The subsequent step involved post-treatment through coagulation-flocculation-sedimentation (CFS), both with and without adsorption on pulverized activated carbon (PAC). This process was conducted at bench scale using jar test equipment (Nova Ética—218 / 6LDBE) under the following operational parameters: rapid mixing time of 1 min, rapid mixing velocity gradient of 600 s^−1^, flocculation mixing time of 20 min, flocculation mixing velocity gradient of 20 s^−1^, settling velocity of 0.25 cm min^−1^, and settling time of 60 min.

The chemicals employed were a commercial ferric chloride solution containing 39.4% FeCl_3_·6H_2_O (mass density = 1.42 kg L^−1^) as a coagulant and hydrochloric acid with 37% HCl (mass density = 1.187 kg L^−1^) as an acidifier.

Preliminary tests supported the determination of the most efficient operating conditions for the physicochemical post-treatment. The optimal coagulant and PAC dosages, as well as the coagulation pH, were established through preliminary bench-scale jar tests conducted under the aforementioned operational parameters. Coagulant dosages ranging from 100 to 800 mg L^−1^, PAC dosages from 500 to 5000 mg L^−1^, and pH values between 3.0 and 10.0 were evaluated.

For the CFS post-treatment, the coagulation condition yielding maximum efficiency was determined to be an Fe dosage of 400 mg L^−1^ at pH 4.0. The effluent generated under these conditions was collected and subjected to ecotoxicity tests.

For the treatment by CFS combined with PAC adsorption, the condition yielding maximum efficiency was as follows: Fe dosage of 200 mg L^−1^ combined with PAC dosage of 500 mg L^−1^ at pH 4.0. The activated carbon was added 1 min after the initiation of chemical coagulation. The PAC used was of mineral origin and chemically activated, characterized by the following parameters: iodine number = 1130 mg g^−1^, methylene blue index = 203 mg g^−1^, and phenol index = 2.2 g L^−1^. The effluent produced under these conditions was also subjected to ecotoxicity tests.

Bench-scale post-treatment assays were carried out using a 2.0 L reaction volume (jar capacity). After sedimentation, the entire supernatant volume (1.0 L) was collected for physicochemical characterization and ecotoxicological bioassays, thereby ensuring representativeness of the evaluated operational conditions.

Except for nitrate analysis, which followed the method proposed by Cataldo et al. (1975), the characterization of the leachate before and after each treatment step was performed using the analytical methodologies outlined in Standard Methods (APHA, 2022). The analyzed parameters included pH, alkalinity, apparent color, true color, Chemical Oxygen Demand (COD), Dissolved Organic Carbon (DOC), Total Kjeldahl Nitrogen (TKN), ammoniacal nitrogen, nitrite, nitrate, chloride, and solids.

### Ecotoxicity Tests

Chronic growth inhibition tests were conducted on *Pseudokirchneriella subcapitata* (Blaise et al. 2000; ABNT, 2023), while acute toxicity tests were performed on *Artemia salina* (Petrobras, 1996; ABNT, 2021), *Daphnia magna* (ABNT, 2022a), and *Ceriodaphnia dubia* (ABNT, 2022b). Ecotoxicity evaluations were carried out using samples of raw leachate (LEACH), leachate treated preliminarily by ammonia stripping followed by biological treatment with activated sludge (BIO), post-treated leachate by coagulation-flocculation-sedimentation (CFS), and post-treated leachate by coagulation-flocculation-sedimentation combined with adsorption on pulverized activated carbon (CFS-PAC).

Prior to the tests, samples of raw leachate and samples from each treatment step were filtered through a cellulose ester membrane with an average pore size of 0.45 µm and stored at − 20 ºC to preserve their characteristics until analysis. The leachate investigated in this study was collected from an old landfill, and its physicochemical characterization indicated parameter values consistent with stabilized leachate, according to Renou et al. (2008), exhibiting recalcitrant characteristics and low biodegradability. Therefore, storage of the samples at − 20 °C was assumed not to significantly influence the results of the toxicity assays.

#### *Artemia salina*

Ecotoxicity tests with *Artemia salina* were conducted in accordance with ABNT NBR 16530:2021 and PETROBRÁS N-2588/1996, with adaptations.

The hatching of *A. salina* cysts (Maramar Aquacultura Com. Imp. Exp. Ltda—ME) involved incubation for 48 h in an artificial saline solution with a pH between 8.0 and 9.0, maintained at a temperature of 27–30ºC under constant lighting. The incubation system consisted of a plastic box compartmentalized by a perforated divider, allowing the nauplii to move by phototropism to the illuminated compartment, while the other compartment was kept dark using aluminum foil.

The tests were performed in 10 mL test tubes, with four replicates for each concentration of leachate samples (limited to 90%), with a total volume of 5 mL, and the pH adjusted to remain between 8.0 and 9.0. Both negative and positive controls were included. After preparing the test tubes with the pre-established concentrations, 10 *A. salina* nauplii were introduced into each tube using a Pasteur pipette with a rounded tip to prevent injury to the organisms. The tubes were then maintained under lighting at a temperature of 27–30 ºC for 24 h.

The negative control was conducted using saline solution, while the positive control used potassium dichromate dissolved in saline solution at a concentration of 0.2 g L^−1^. After 24 h of exposure, the number of live and dead organisms in each tube was recorded. From these data, the concentration of the sample that caused 50% lethality or immobility of the organisms after 24 h of exposure (LC50_24h_) was determined using the Trimmed Spearman-Karber method (Hamilton et al., 1977), with a 95% confidence interval.

The test was considered valid if the percentage of immobile or dead organisms was below 10% in the negative control and above 90% in the positive control (ABNT, 2021).

#### *Daphnia magna*

Ecotoxicity tests with *Daphnia magna* were performed following the ABNT NBR 12713:2022 standard, with adaptations.

The tests involved exposing five neonates aged between 2 and 26 h to different dilutions of the leachate samples in culture water (M4 medium) with a total volume of 10 mL, contained in polypropylene cell culture plates. Each sample concentration and the negative control (M4 medium) were tested in triplicate. The experiments were conducted at a controlled temperature of 22ºC, without lighting and feeding.

After the exposure period, immobilized and dead organisms were counted using a stereoscope (Motic—SMZ140 FBLED). The results were expressed as the median effective concentration of the sample that affected 50% of the exposed population after 24 h (EC50_24h_) and 48 h (EC50_48h_). The EC50 values were calculated using the Trimmed Spearman-Karber method (Hamilton et al., 1977) with a 95% confidence interval.

The test was considered valid only if the percentage of immobile or dead organisms in the negative control was below 10% (ABNT, 2022a).

#### *Ceriodaphnia dubia*

Ecotoxicity tests with *Ceriodaphnia dubia* were conducted in accordance with the ABNT NBR 13373:2022 standard, with adaptations.

The tests involved exposing five neonates aged between 6 and 24 h to different dilutions of the leachate samples in reconstituted water, with a total volume of 10 mL, contained in polypropylene cell culture plates. Each sample concentration and the negative control (reconstituted water) were tested in triplicate. The experiments were carried out at a controlled temperature of 22ºC, without lighting and feeding.

Similar to the tests with *Daphnia magna*, immobilized and dead organisms were counted after the exposure period using a stereoscope. The results were expressed as the median effective concentration for 24 h (EC50_24h_) and 48 h (EC50_48h_), obtained through statistical analysis using the Trimmed Spearman–Karber method with a 95% confidence interval. The tests were considered valid only if the percentage of immobile or dead organisms in the negative control was below 10% (ABNT, 2022b).

#### *Pseudokirchneriella subcapitata*

The organism *Pseudokirchneriella subcapitata* is standardized by ABNT NBR 12648:2023. The protocol used for the toxicity tests with *P. subcapitata* was based on the methodology described by Blaise et al. (2000). Test validity was conditioned on the following criteria: the coefficient of variation among five control samples, with an exposure time of 72 h, could not exceed 40%, and the cell density in the control flasks had to increase by a factor of at least 16 (1.16 × 10^5^ cells mL^−1^).

Different concentrations of the leachate samples, diluted in a sodium bicarbonate buffer solution, were prepared in 5 mL vials with a total volume of 2.5 mL, with five replicates for each concentration. Estimated populations of *P. subcapitata* of approximately 1.04 × 10^4^ cells mL^−1^ were exposed in these vials. The flasks were sealed with transparent plastic film and incubated under continuous light at 25 °C for 72 h in a shaker (Tecnal—TE141).

After the incubation period, toxicity was assessed by determining the percentage of inhibition, with cell counts conducted using an optical microscope (Motic-BA210) and a Neubauer chamber. The results were analyzed using the Trimmed Spearman-Karber statistical method (Hamilton et al., 1977) with a 95% confidence interval and expressed as IC50_72h_, representing the mean effective concentration causing a chronic effect to 50% of the organisms after 72 h of exposure.

## Results and Discussion

### Leachate Characterization

Table 1 presents the results of the physical and chemical characterization of the raw leachate (LEACH), the leachate treated by ammonia stripping followed by activated sludge treatment (BIO), and the leachate post-treated through coagulation-flocculation-sedimentation (CFS) and adsorption with pulverized activated carbon (CFS-PAC), under the selected conditions described in the previous section.

**Table 1:** Physicochemical characterization of raw leachate samples and samples from the sequential treatment stages

Parameter	Unity	Leachate samples			
		LEACH	BIO	CFS	CFS-PAC
pH	–	8.9	8.5	4.1	4.1
Alkalinity	mg CaCO_3_ L^−1^	1914	1608	–	–
Apparent color	HU	4031	3504 (13.1%)*	75 (98.1%)*	–
True color	HU	3598	3393 (5.7%)*	38 (98.9%)*	16 (99.5%)*
COD	mg O_2_ L^−1^	1344	1144 (14.9%)*	292 (78.3%)*	183 (86.4%)*
DOC	mg C L^−1^	685	672 (1.9%)*	23 (96.6%)*	32 (95.3%)*
TKN	mg N-NH_3_ L^−1^	304	67 (78.0%)*	15 (95.1%)*	–
N-amoniacal	mg N-NH_3_ L^−1^	236	5 (97.9%)*	2 (99.1%)*	–
Nitrite	mg N-NO_2_ L^−1^	105	3 (97.1%)*	ND**	–
Nitrate	mg N-NO_3_ L^−1^	21	ND**	ND**	–
Chloride	mg L^−1^	1828	1935	3161	3111
TS	mg L^−1^	5554	6456	6330	–
TSS	mg L^−1^	232	73	22	–
TDS	mg L^−1^	5322	6383	6308	–

^*^Removal percentages in parentheses

^**^N.D = Not detected (LOD_NITRITE_ = 0.007 mg L^−1^; LOD_NITRATE_ = 3.3 mg L^−1^)

According to Renou et al. (2008), stabilized leachates are characterized by pH values higher than 7.5 and COD concentrations lower than 4000 mg L^−1^. The leachate investigated in this study exhibited characteristics consistent with the biological stabilization phase, presenting a pH of 8.9 and a COD concentration of 3598 mg L^−1^, thus indicating low biodegradability.

The preliminary treatment by ammonia stripping followed by biological treatment with activated sludge showed high removal efficiency for nitrogenous compounds, achieving 78.0% removal of TKN, 97.9% of ammoniacal nitrogen, 97.1% of nitrite, and > 84.3% of nitrate. However, the biologically treated leachate did not exhibit significant removal of recalcitrant organic matter, with residual values of true color, COD, and DOC of 3393 HU (5.7% removal), 1144 mg L^−1^ (14.9% removal), and 672 mg L^−1^ (1.9% removal), respectively, confirming its low biodegradability.

In the post-treatment using chemical coagulation-flocculation-sedimentation, the selected condition (Fe dosage of 400 mg L^−1^ at pH 4) produced a decanted leachate with residual values of 38 HU for true color (98.9% removal), 292 mg L^−1^ for COD (78.3% removal), and 23 mg L^−1^ for DOC (96.6% removal).

When coagulation-flocculation-sedimentation was combined with adsorption on pulverized activated carbon, the selected condition (Fe dosage of 200 mg L^−1^ and PAC dosage of 500 mg L^−1^, at pH 4.0) yielded a higher-quality effluent, with residual values of 16 HU for true color (99.5% removal), 183 mg L^−1^ for COD (86.4% removal), and 32 mg L^−1^ for DOC (95.3% removal).

The combination of coagulation-flocculation-sedimentation with adsorption on pulverized activated carbon was identified as the most efficient technical alternative for post-treatment. This assessment considered the effluent quality concerning recalcitrant organic matter (COD and true color), chemical costs, and feasibility for full-scale applications.

### Ecotoxicity Tests

**Fig. 1:**
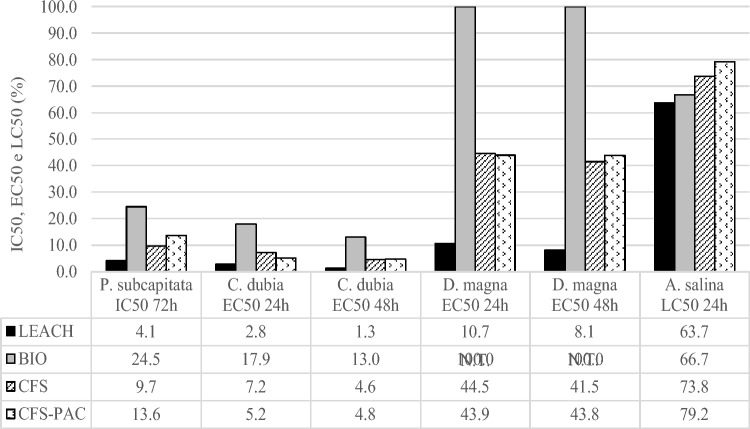
Results of ecotoxicity tests for various organisms and leachate samples. N.T, Non-toxic

Figure 1 presents the results of the ecotoxicity tests, expressed as IC50_72h_ for *P. subcapitata*, EC50_24h_ and EC50_48h_ for *C. dubia* and *D. magna*, and LC50_24h_ for *A. salina*, corresponding to the samples of raw leachate (LEACH), leachate following preliminary treatment by ammonia stripping and biological treatment via activated sludge (BIO), post-treated leachate using coagulation-flocculation-sedimentation (CFS), and post-treated leachate by CFS combined with PAC adsorption (CFS-PAC). All ecotoxicological assays met the respective validity criteria established for each test organism. In general, mortality in the negative controls remained below 10%, while the positive controls showed responses above 90%, confirming the reliability of the experimental results.

Considering the results obtained for IC50_72h_ in *P. subcapitata* and EC50_48h_ in *C. dubia* for the LEACH and BIO samples, a significant reduction in toxicity was observed, with concentration values increasing from 4.1 to 24.5 for *P. subcapitata* and from 1.3 to 13.0 for *C. dubia*.

For *D. magna*, the EC50_24h_ and EC50_48h_ values for the raw leachate were 10.7 and 8.1%, respectively. Gotvan et al. (2009) observed EC50_24h_ and EC50_48h_ values of 3.7 and 3.2%, respectively, for raw leachate in *D. magna*. Comparable results were also documented by Martins et al. (2010), who reported EC50_48h_ values ranging from 2.28 to 3.12%; by Da Silva (2022), who obtained an EC50_48h_ value of 1.22%; and by Fernandes et al. (2019), who reported similar values close to 1%, further highlighting the high toxicity of the effluent to *D. magna*.

However, no toxicity was detected in the leachate following biological treatment, making it impossible to determine EC50_24h_ and EC50_48h_ for *D. magna* in the treated samples. This outcome is likely attributable to the high concentration of nitrogen in the form of ammonia (236 mg L^−1^) in the raw leachate, which is toxic to organisms, and its subsequent 97.9% removal after biological treatment. The results obtained by Costa et al. (2023) also indicate that the high concentration of ammoniacal nitrogen in the leachate significantly contributes to its toxicity. These findings highlight the effectiveness of biological treatment in reducing toxicity. Similar findings were reported by Kawahigashi (2012), where the toxicity of raw leachate decreased from concentration values between 0.9 and 4.7% to values between 20.6 and 30.1% following biological treatment.

Despite the effectiveness of post-treatments using CFS and CFS-PAC in removing apparent and true color, as well as COD (Table 1), an increase in leachate toxicity was observed. For *D. magna*, which exhibited no immobility or lethality effects after biological treatment, EC50_48h_ values of 41.5 and 43.8% were recorded for CFS and CFS-PAC, respectively. A similar trend was observed for *P. subcapitata*, with IC50_72h_ values of 9.7 and 13.6%, and for *C. dubia*, with EC50_48h_ values of 4.6 and 4.8%, respectively.

This increase in toxicity may be attributed to compounds derived from the chemicals used in the coagulation process, such as iron (Fe), chlorides, or other by-products introduced by the coagulant (ferric chloride) during post-treatment. However, further investigations are required (e.g., conducting bioassays with the test organisms using different concentrations of residual metals and chlorides) to substantiate this hypothesis.

The increase in toxicity observed after CFS and CFS–PAC treatment may have important practical implications for the management of leachate treatment plants. Although these processes are effective in reducing parameters associated with recalcitrant organic matter (true color, COD, and DOC), the ecotoxicological results indicate that improvements in physicochemical characteristics do not necessarily correspond to a reduction in biological risk. From a regulatory perspective, this finding highlights the importance of integrating ecotoxicity assays into the routine monitoring of treatment plants, since compliance based solely on conventional physicochemical parameters may be insufficient, particularly when physicochemical polishing steps are applied. Operationally, these results suggest the need for careful optimization of coagulant dosage and, if necessary, the inclusion of additional polishing steps to ensure that the final effluent meets environmental safety standards.

In contrast to the responses observed in other test organisms, the toxicity of the raw leachate for *A. salina* showed a gradual reduction following each treatment step. The LC50_24h_ values recorded were 63.7, 66.7, 73.8, and 79.2 for the LEACH, BIO, CFS, and CFS-PAC samples, respectively.

These results suggest that the sensitivity of *A. salina* to recalcitrant organic matter in stabilized leachate may differ from that of the other tested organisms. This difference could be related to its marine origin and a potentially higher tolerance to chlorides, which may be toxic to *D. magna* and *C. dubia*, both freshwater organisms. Consequently, *A. salina* may represent a more suitable indicator organism for ecotoxicity tests involving stabilized leachate, although further investigation is required to confirm this interpretation.

**Fig. 2:**
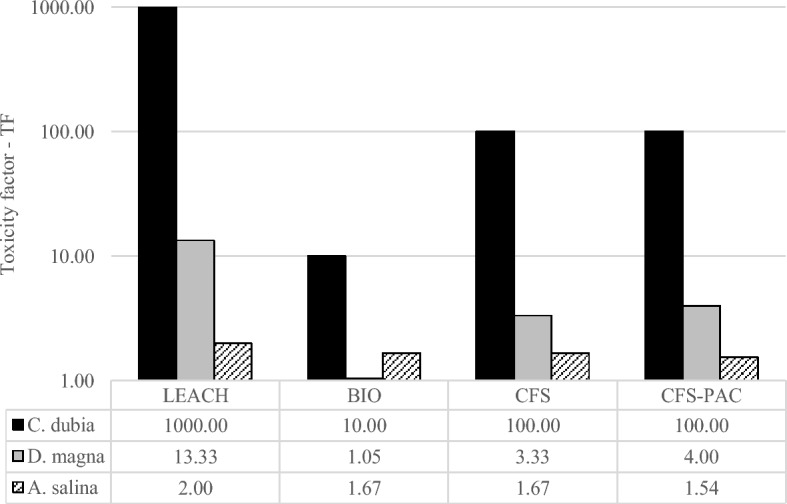
Toxicity factor (TF) values for different test organisms and leachate samples

Figure 2 presents the toxicity factor (TF) values obtained, defined as the lowest dilution of the sample at which no harmful effects on the test organisms (*C. dubia*, *D. magna*, and *A. salina*) are observed. According to Resolution No. 081/2010 of CEMA—State Environmental Council, Paraná, Brazil (PARANÁ, 2010), criteria and standards for acute toxicity are established for controlling liquid effluents discharged into surface waters. This resolution limits the TF value to 8 (12.5%) for *Daphnia magna* and *Vibrio fischeri* in effluents released into freshwater bodies.

Analyzing the TF results for *D. magna*, it is evident that the factor exceeded 8 for the raw leachate. However, following biological treatment, the TF value decreased to 1.05, remaining within the established limit. For the leachates treated by CFS and CFS-PAC, TF values of 3.33 and 4, respectively, also comply with the limit established by the resolution.

The sensitivity of the test organisms in the ecotoxicity tests varied depending on the compounds present in the leachate. This variability is attributed to their distinct morphological characteristics and habitats. Consequently, to comprehensively assess the toxic effects of a sample, it is recommended to conduct ecotoxicity tests using organisms from different trophic levels of the food chain. These may include microalgae, microcrustaceans, echinoids, polychaetes, oligochaetes, fish, and bacteria, thereby encompassing a broad range of ecosystems and trophic levels.

## Conclusions

The ecotoxicity tests, represented by IC50_72h_ for *P. subcapitata*, EC50_24h_ and EC50_48h_ for *C. dubia* and *D. magna*, and LC50_24h_ for *A. salina*, demonstrated that biologically treated leachate exhibited lower toxicity for the tested organisms compared to raw leachate. This result highlights the efficiency of ammonia stripping followed by activated sludge treatment in reducing contaminants that contribute to toxicity.

In the post-treatment by coagulation–flocculation–sedimentation (CFS), despite the substantial removal of recalcitrant compounds, toxicity increased for *P. subcapitata*, *C. dubia*, and *D. magna*. This increase in toxicity may be associated with residual metals and chlorides derived from the coagulant (ferric chloride) used during the process. However, further investigations are required to better substantiate this hypothesis.

Considering the experimental conditions and the limitations of this study, *A. salina* exhibited responses that differed from those of the other test organisms. For this species, the toxicity of the raw leachate tended to decrease after each treatment stage. This behavior may be associated with its marine origin and potential tolerance to chlorides, a compound that may affect freshwater organisms such as *P. subcapitata*, *C. dubia*, and *D. magna*. These findings suggest that *A. salina* may respond more consistently to toxicity reductions associated with recalcitrant organic matter in stabilized leachates, since chloride presence may exert less influence on its responses; however, this interpretation should be considered with caution and supported by further studies.
